# 171. The Impact of COVID-19 on Healthcare-Associated Infections

**DOI:** 10.1093/ofid/ofab466.171

**Published:** 2021-12-04

**Authors:** Meghan A Baker, Kenneth Sands, Susan S Huang, Ken Kleinman, Edward Septimus, Neha Varma, Eunice J Blanchard, Russell Poland, Micaela H Coady, Deborah S Yokoe, Deborah S Yokoe, Sarah Fraker, Allison Froman, Julia Moody, Laurel Goldin, Amanda Isaacs, Kacie Kleja, Kimberly Korwek, John Stelling, Adam Clark, Richard Platt, Jonathan B Perlin

**Affiliations:** 1 Harvard Medical School/Harvard Pilgrim Health Care Institute and Brigham and Women’s Hospital, Boston, Massachusetts; 2 HCA Healthcare, Nashville, TN; 3 University of California, Irvine, Irvine, CA; 4 University of Massachusetts, Amherst, Massachusetts; 5 Harvard Medical School, Houston, Texas; 6 Harvard Pilgrim Health Care Institute, boston, Massachusetts; 7 University of California, San Francisco, San Francisco, CA; 8 Brigham and Women’s Hospital, Boston, Massachusetts

## Abstract

**Background:**

The profound changes wrought by COVID-19 on routine hospital operations may have influenced performance on hospital measures, including healthcare-associated infections (HAIs).

**Objective:**

Evaluate the association between COVID-19 surges and HAI or cluster rates

**Methods:**

**Design:** Prospective cohort study

**Setting:**

148 HCA Healthcare-affiliated hospitals, 3/1/2020-9/30/2020, and a subset of hospitals with microbiology and cluster data through 12/31/2020

**Patients:**

All inpatients

**Measurements:**

We evaluated the association between COVID-19 surges and HAIs, hospital-onset pathogens, and cluster rates using negative binomial mixed models. To account for local variation in COVID-19 pandemic surge timing, we included the number of discharges with a laboratory-confirmed COVID-19 diagnosis per staffed bed per month at each hospital.

**Results:**

Central line-associated blood stream infections (CLABSI), catheter-associated urinary tract infections (CAUTI), and methicillin-resistant *Staphylococcus aureus* (MRSA) bacteremia increased as COVID-19 burden increased (P ≤ 0.001 for all), with 60% (95% CI, 23 to 108%) more CLABSI, 43% (95% CI, 8 to 90%) more CAUTI, and 44% (95% CI, 10 to 88%) more cases of MRSA bacteremia than expected over 7 months based on predicted HAIs had there not been COVID-19 cases. *Clostridioides difficile* infection (CDI) was not significantly associated with COVID-19 burden. Microbiology data from 81 of the hospitals corroborated the findings. Notably, rates of hospital-onset bloodstream infections and multidrug resistant organisms, including MRSA, vancomycin-resistant enterococcus and Gram-negative organisms were each significantly associated with COVID-19 surges (P < 0.05 for all). Finally, clusters of hospital-onset pathogens increased as the COVID-19 burden increased (P = 0.02).

**Limitations:**

Variations in surveillance and reporting may affect HAI data.

Table 1. Effect of an increase in number of COVID-19 discharges on HAIs and hospital-onset pathogens

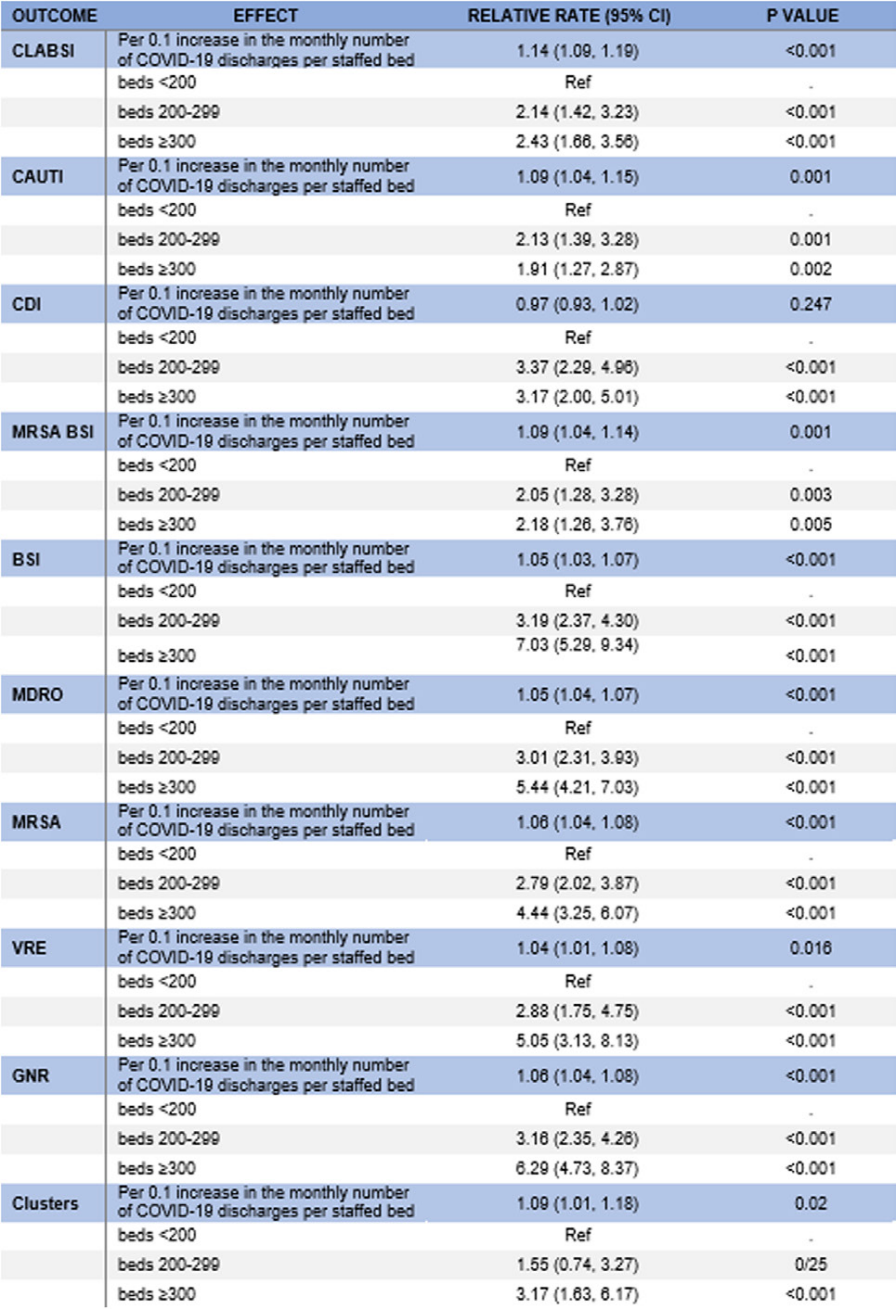

Figure 1. Predicted mean HAI rates as COVID-19 discharges increase

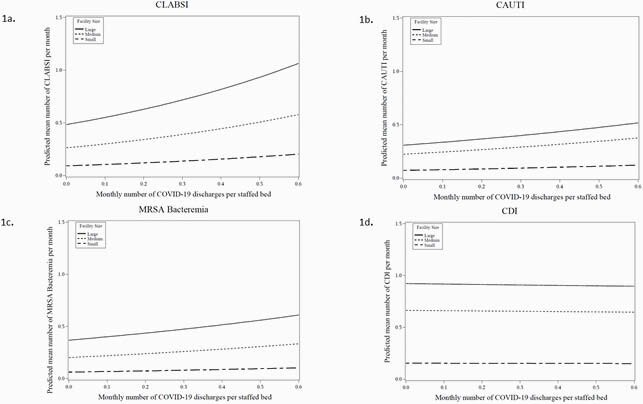

Predicted mean HAI rate by increasing monthly COVID-19 discharges. Panel a. CLABSI, Panel b, CAUTI Panel c. MRSA Bacteremia, Panel d. CDI. Data are stratified by small, medium and large hospitals.

Figure 2. Monthly comparison of COVID discharges to clusters

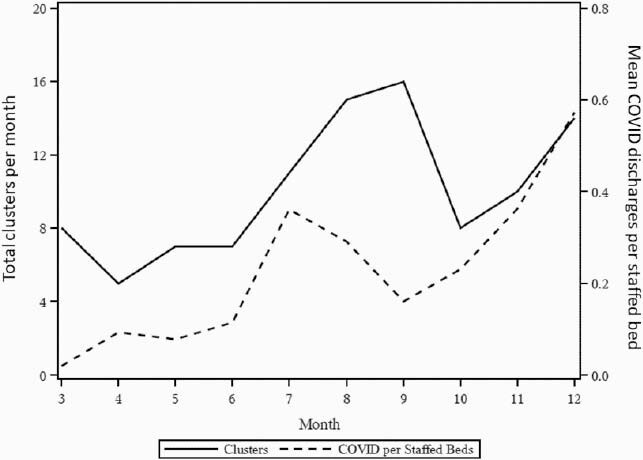

COVID-19 discharges and the number of clusters of hospital-onset pathogens are correlated throughout the pandemic.

**Conclusion:**

COVID-19 surges adversely impact HAI rates and clusters of infections within hospitals, emphasizing the need for balancing COVID-related demands with routine hospital infection prevention.

**Disclosures:**

**Kenneth Sands, MD, MPH**, **Medline** (Other Financial or Material Support, Conducted studies in which participating hospitals received contributed antiseptic product) **Susan S. Huang, MD, MPH**, **Medline** (Other Financial or Material Support, Conducted studies in which participating hospitals and nursing homes received contributed antiseptic and cleaning products)**Molnlycke** (Other Financial or Material Support, Conducted studies in which participating hospitals and nursing homes received contributed antiseptic and cleaning products)**Stryker (Sage**) (Other Financial or Material Support, Conducted studies in which participating hospitals and nursing homes received contributed antiseptic and cleaning products)**Xttrium** (Other Financial or Material Support, Conducted studies in which participating hospitals and nursing homes received contributed antiseptic and cleaning products) **Ken Kleinman, PhD**, **Medline** (Other Financial or Material Support, Conducted studies in which participating hospitals received contributed antiseptic products)**Molnlycke** (Other Financial or Material Support, Conducted studies in which participating hospitals received contributed antiseptic products) **Edward Septimus, MD**, **Medline** (Other Financial or Material Support, Conducted studies in which participating hospitals received contributed antiseptic products)**Molnlycke** (Other Financial or Material Support, Conducted studies in which participating hospitals received contributed antiseptic products) **Eunice J. Blanchard, MSN RN**, **Medline** (Other Financial or Material Support, Conducted studies in which participating hospitals received contributed antiseptic product) **Russell Poland, PhD**, **Medline** (Other Financial or Material Support, Conducted studies in which participating hospitals received contributed antiseptic product) **Micaela H. Coady, MS**, **Medline** (Other Financial or Material Support, Conducted studies in which participating hospitals received contributed antiseptic product)**Molnlycke** (Other Financial or Material Support, Conducted studies in which participating hospitals received contributed antiseptic product) **Deborah S. Yokoe, MD, MPH**, Nothing to disclose **Julia Moody, MS**, **Medline** (Other Financial or Material Support, Conducted studies in which participating hospitals received contributed antiseptic product)**Molnlycke** (Other Financial or Material Support, Conducted studies in which participating hospitals received contributed antiseptic product) **Richard Platt, MD, MSc**, **Medline** (Research Grant or Support, Other Financial or Material Support, Conducted studies in which participating hospitals received contributed antiseptic product)**Molnlycke** (Other Financial or Material Support, Conducted studies in which participating hospitals received contributed antiseptic product) **Jonathan B. Perlin, MD, PhD**, **Medline** (Other Financial or Material Support, Conducted studies in which participating hospitals received contributed antiseptic product)**Molnlycke** (Other Financial or Material Support, Conducted studies in which participating hospitals received contributed antiseptic product)

